# Passive Dielectrophoretic Focusing of Particles and Cells in Ratchet Microchannels

**DOI:** 10.3390/mi11050451

**Published:** 2020-04-25

**Authors:** Song-Yu Lu, Amirreza Malekanfard, Shayesteh Beladi-Behbahani, Wuzhou Zu, Akshay Kale, Tzuen-Rong Tzeng, Yao-Nan Wang, Xiangchun Xuan

**Affiliations:** 1 Department of Mechanical Engineering, Clemson University, Clemson, SC 29634-0921, USA; songyul@g.clemson.edu (S.-Y.L.); amaleka@g.clemson.edu (A.M.); wzu@g.clemson.edu (W.Z.); 2 Department of Vehicle Engineering, National Pingtung University of Science and Technology, Pingtung 912, Taiwan; 3 Department of Biological Sciences, Clemson University, Clemson, SC 29634-0314, USA; sbeladi@g.clemson.edu (S.B.-B.); tzuenrt@clemson.edu (T.-R.T.); 4 Electrical Engineering Division, CAPE Building, Department of Engineering, University of Cambridge, 9 JJ Thomson Avenue, West Cambridge Site, Cambridge CB3 0FA, UK; ak2115@cam.ac.uk

**Keywords:** electrokinetic, dielectrophoresis, particle focusing, microfluidics

## Abstract

Focusing particles into a tight stream is critical for many microfluidic particle-handling devices such as flow cytometers and particle sorters. This work presents a fundamental study of the passive focusing of polystyrene particles in ratchet microchannels via direct current dielectrophoresis (DC DEP). We demonstrate using both experiments and simulation that particles achieve better focusing in a symmetric ratchet microchannel than in an asymmetric one, regardless of the particle movement direction in the latter. The particle focusing ratio, which is defined as the microchannel width over the particle stream width, is found to increase with an increase in particle size or electric field in the symmetric ratchet microchannel. Moreover, it exhibits an almost linear correlation with the number of ratchets, which can be explained by a theoretical formula that is obtained from a scaling analysis. In addition, we have demonstrated a DC dielectrophoretic focusing of yeast cells in the symmetric ratchet microchannel with minimal impact on the cell viability.

## 1. Introduction

Microfluidic devices have been widely used to handle (e.g., focus [1], count [2], trap [3], and sort [4] etc.) various types of particle for biomedical, chemical, and environmental applications. Focusing particles into a tight stream is critical to many of these particle-handling devices such as flow cytometers [5,6] and particle sorters [7,8,9,10]. Sheath fluids are often used to confine particles into a well-defined volume, which, however, requires an accurate control of flow rates. This is because sheath-flow focusing acts upon the suspending fluid, not the suspended particles [11]. Therefore, a variety of forces, which may be externally imposed (termed as *active* focusing) or internally induced (termed as *passive* focusing), has been demonstrated to directly manipulate particles for sheath-free focusing [12]. For the *active* focusing of particles, the application of an external acoustic [13], alternating current (AC) electric [14], or magnetic [15] field creates a non-invasive force that drives particles across fluid streamlines. This type of method requires an additional field source other than that pumping the particle suspension, not mentioning the other added difficulties such as the patterning of microelectrodes for acoustic [16] or dielectrophoretic [17] focusing and the magnetic labeling of typically non-magnetic particles [18]. The *passive* focusing of particles relies on a flow- and/or a channel structure-induced transverse force to direct particles towards one or multiple equilibrium positions over the channel cross-section. This type of method requires only one external field source to generate the flow of the particle suspension wherein the particles are automatically focused without any other controls. It is therefore easy to operate and ready to be integrated with a pre- and/or a post-focusing component for lab-on-a-chip systems [12].

Among the flow-induced *passive* particle focusing methods, inertial focusing has been rapidly growing since the seminal work of Di Carlo et al. [19]. It exploits the fluid inertia-induced lift force to focus particles down to multiple or even single streams at high throughput [20,21,22,23]. Elastic focusing results from the fluid rheology-induced lift force that is capable of manipulating much smaller particles than inertial focusing [24,25,26,27]. The combination of elastic and inertial focusing can further enhance the particle control [28] and extend the working range of flow rates [29]. Among the channel structure-induced *passive* particle focusing methods, hydrophoretic focusing utilizes the anisotropic fluid resistance of slant obstacles to generate transverse flows that carry particles towards the sidewall or channel center [30]. Hydrodynamic filtration-based focusing is based on the split and recombination of fluid flows in multiple loop channels that are symmetrically arranged on both sides of the main microchannel [31]. In addition, a direct current (DC) electric field has been demonstrated to both electrokinetically transport (via fluid electroosmosis and particle electrophoresis) and passively focus particles in a straight uniform microchannel via wall-induced electrical lift [32]. Moreover, its gradient can induce particle dielectrophoresis (DEP) for *passive* focusing in either a straight microchannel with a varying cross-section [33] or a curved microchannel [34]. The so-called insulator-based dielectrophoresis (iDEP) in the former case has been extensively demonstrated to trap [35,36], pattern [37], electroporate [38], and separate [39,40,41,42,43] particles in a continuous electrokinetic flow under either a DC or a DC-biased AC electric field. The effects of insulator structure, electric field, particle properties (e.g., size, charge, and type), and surface treatment have all been investigated [44,45,46]. 

However, there has been much less work on particle focusing in iDEP microdevices. A DC-biased AC electric field is necessary for the focusing of particles in a single-constriction microchannel [47], which is an *active* focusing method because the DC component pumps the particle suspension while the AC component supplements particle DEP. The *passive* focusing of particles under a DC electric field has been demonstrated in a single-constriction microchannel only when the size of the constriction closely matches that of the particles [48] or the channel-to-constriction area ratio becomes very large [33]. It can also be realized by the use of an array of ratchets, which, as reported in this work, forms periodic constrictions for a significantly extended working range of DEP. We perform a combined experimental, numerical, and theoretical study of the effects of ratchet structure, electric field, and particle size on the DC dielectrophoretic focusing of particles in ratchet microchannels. We also demonstrate the biological application of this *passive* particle focusing method to yeast cells.

## 2. Experiment

### 2.1. Materials

**Figure 1:**
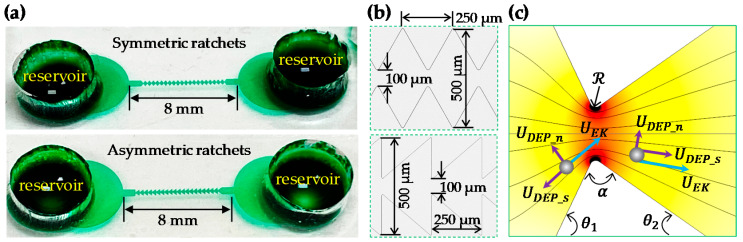
(**a**) Photos of the symmetric (top) and asymmetric (bottom) ratchet microchannels used in the experiment; (**b**) Zoomed-in views of the symmetric (top) and asymmetric (bottom) ratchet structures with their corresponding dimensions highlighted; (**c**) Velocity analysis for a particle traveling towards and away from the ratchet throat, respectively, where the background color shows the electric field contour (the darker, the larger magnitude) and the background lines represent the electric field lines (equivalent to the fluid streamlines).

Two types of ratchet microchannel were used in this work, which, as shown in Figure 1a, were composed of 20 consecutive symmetric and asymmetric ratchets, respectively. They were fabricated with polydimethylsiloxane (PDMS) using the standard soft lithography technique. The broadest part of the microchannel was 500 μm wide and the narrowest part between the opposing ratchet tips was 100 μm wide in both channel structures (see the zoomed-in views in Figure 1b). The period at which the ratchet structure repeats itself, i.e., the peak-to-peak distance of two consecutive ratchets, is 250 μm, leading to an overall 5 mm long ratchet region. The total length of each ratchet microchannel is 8 mm, and the depth is uniformly 40 μm. Spherical polystyrene particles of 3, 5, and 10 μm diameter (Sigma-Aldrich Corp., St. Louis, MO, USA) were re-suspended in 1 mM phosphate buffer solution with a measured electric conductivity of 200 µS/cm (Fisher Scientific, Accumet AP85, Waltham, MA, USA). ATCC9763 yeast cells (Saccharomyces cerevisiae) were cultured at 35 °C in Sabouraud dextrose broth (Becton and Dickinson Co., Franklin Lakes Township, NJ, USA) medium. They were harvested after 24 h and washed three times with phosphate buffered saline (PBS) solution. Prior to use, yeast cells were re-suspended in 1 mM phosphate buffer to a final concentration of around 10^5^ cells/mL. They were measured to have an average diameter of around 5 μm. To avoid particle/cell aggregations and adhesions (to microchannel walls), a small amount of Tween 20 (0.5 % v/v, Fisher Scientific, Waltham, MA, USA) was added into each suspension.

### 2.2. Methods

The DC electric field across the ratchet microchannels was generated by a high-voltage DC power supply (Glassman High Voltage Inc., High Bridge, NJ, USA) via platinum electrodes. To avoid Joule heating effects [49], the average field magnitude was kept no more than 500 V/cm (i.e., a 400 V voltage drop over the 0.8 cm long microchannel) in all tests. Prior to every test, the liquid heights in the two reservoirs were carefully balanced to eliminate the flow due to hydrostatic pressure. Moreover, the time of the application of the electric field was limited to no more than 2 min in order to minimize the electroosmosis-induced pressure-driven backflow [50]. Each test was repeated at least three times on different days to ensure the repeatability of the attained results. The motions of particles and cells at different locations of the microchannel were captured using an inverted microscope (Nikon Eclipse TE2000U; Nikon Instruments, Lewisville, TX, USA) with a CCD Camera (Nikon DS-Qi1Mc, Lewisville, TX, USA) at a rate of around 15 frames per second. The obtained digital images were post-processed in the Nikon imaging software (NIS-Elements AR 2.30, Lewisville, TX, USA). The electrokinetic mobility (= electrokinetic velocity/electric field) of the particles was determined by measuring the particle velocity in the region away from the ratchets where the particle DEP was negligible. We found an approximately identical mobility of 1.86 × 10^−8^ m^2^/(V⋅s) for all three sizes of particle used in the experiment. 

## 3. Theory

### 3.1. Focusing Mechanism

The insulating ratchets create electric field gradients around them (see the contour in Figure 1c) in a microchannel because of: (1) the variation in the cross-sectional area from the channel to the constriction formed by the facing ratchets, which is primarily along the direction of the electric field lines (or equivalently, the fluid streamlines because of their similarity in purely electrokinetic flows under the thin electric double layer assumption [51]); and (2) the variation in the path length for electric current around the ratchet tips, which is primarily normal to the direction of electric field lines. Thus, a dielectrophoretic force is induced by the ratchets, which acts on the suspended particles and cells. As they are less conductive than the suspending medium in our experiment, the polystyrene particles and yeast cells tend to be pushed away from the regions with a higher electric field, i.e., the ratchet tip (see Figure 1c), by negative DEP. Therefore, particles get focused towards the centerline of the microchannel when they travel through the ratchet region electrokinetically. Such a focusing effect via DC DEP can be characterized by the (dimensional) particle deflection that depends on the ratio of the normal component (i.e., perpendicular to the electric field lines in Figure 1c) of the particle velocity to the streamwise component (i.e., tangential to the electric field lines) within one period of the ratchets:

(1) $$deflection=\frac{\left|U_{DEP\_n}\right|ℛ\alpha }{\left|U_{EK}+U_{DEP\_s}\right|}$$

where $U_{DEP}$ is the dielectrophoretic particle velocity, with the subscripts n and s denoting, respectively, the normal and stream-wise directions; $U_{EK}$ is the streamwise electrokinetic velocity; and the product ℛα measures the working distance for the cross-stream particle DEP, with ℛ and α being the curvature radius and opening angle (in the unit of radians) of the ratchet tip (see Figure 1c), respectively. Note that velocity magnitudes are used in Equation (1) because both $U_{DEP}$ and $U_{EK}$ can be positive or negative. It is also important to point out that the particle deflection in Equation (1) is not a constant because both $U_{DEP}$ and $U_{EK}$ vary with the particle position. 

Following the traditional analysis of electrokinetic phenomena [52], the particle deflection in Equation (1) may be rewritten as

(2) $$deflection=\frac{\left|\mu_{DEP}\nabla_{n}E^{2}\right|ℛ\alpha }{\left|\mu_{EK}E+\mu_{DEP}\nabla_{s}E^{2}\right|}=\frac{ℛ\alpha \left|\mu_{DEP}\frac{2E^{2}}{ℛ}\right|}{\left|\mu_{EK}E+\mu_{DEP}\frac{\partial E^{2}}{\partial s}\right|}=\frac{2\alpha }{\left|\frac{\mu_{EK}}{\mu_{DEP}}\frac{1}{E}+\frac{2}{E}\frac{\partial E}{\partial s}\right|}$$

(3) $$\mu_{DEP}=f_{CM}\frac{d^{2}\varepsilon }{12\eta }$$

where $\mu_{DEP}$ is the dielectrophoretic particle mobility, $\mu_{EK}$ is the electrokinetic particle mobility, and E is the electric field magnitude. In the definition of $\mu_{DEP}$, $f_{CM}=\left(\sigma_{p}-\sigma \right)/\left(\sigma_{p}+2\sigma \right)$ is the Clausius–Mosotti factor, with $\sigma_{p}$ and σ being the particle and fluid electric conductivities, respectively; d is the (spherical) particle diameter; ε is the fluid electric permittivity; and η is the fluid viscosity. As illustrated by the particle velocity analysis in Figure 1c, the streamline component of the dielectrophoretic particle velocity, $U_{DEP\_s}$, slows down the electrokinetic particle motion towards the ratchet throat while accelerating it when the particle is traveling away. Its impact on the particle deflection hence becomes a strong function of the ratchet structure as determined by the angles $\theta_{1}$ and $\theta_{2}$ (note these two angles are dependent on each other if the height and width of each ratchet are both fixed). Moreover, as $\alpha =\pi -\theta_{1}-\theta_{2}$ (see Figure 1c), the impact of the normal component of the dielectrophoretic particle velocity, $U_{DEP\_n}$, on the particle focusing effect is also a function of the ratchet structure. In addition, Equation (2) predicts an enhanced deflection for larger particles at a higher electric field. All these effects are examined in this work. It is interesting to see that the particle deflection in Equation (2) becomes independent of the curvature radius of the ratchet tip. This is because we assume that particles traveling around the ratchet behave like those traveling through an exactly circular channel [52]. 

### 3.2. Numerical Modeling

A two-dimensional numerical model was developed in COMSOL^®^ Multiphysics 5.3a to understand and simulate the observed particle focusing effect in the tested two-dimensional ratchet microchannels. A Lagrangian tracking method was used to trace the motion of particles in the electric field-driven fluid flow under various conditions [53]. Only the electric field was solved using the “Electric Currents (ec)” module because of the similarity between the electric field lines and fluid streamlines in purely electrokinetic flows [51]. Particle trajectories were plotted using the particle tracing function in COMSOL^®^ via the particle velocity, $U_{P}$, which, as shown in Figure 1c, is the vector sum of the electrokinetic and dielectrophoretic velocities:

(4) $$U_{P}=U_{EK}+\lambda U_{DEP}=\mu_{EK}E+\lambda \mu_{DEP}\nabla E^{2}$$

where E is the electric field vector and λ is the correction factor that accounts for the effect of particle size on the dielectrophoretic velocity [54]. It is because the particle’s disturbances to the electric field (and as well the flow field) were neglected in the model. Such a treatment has been proved effective in our earlier studies as well as in those from other research groups [55]. To calculate the Clausius–Mosotti factor, $f_{CM}$, in Equation (3), we assumed that the electric conductivity of polystyrene particles is determined solely by the surface conduction, $\sigma_{s}=1$ nS, through $\sigma_{p}=4\sigma_{s}/d$ [56]. The obtained values are hence −0.45, −0.47, and −0.49 for 3, 5, and 10 µm particles, respectively. The fluid permittivity and viscosity were both assumed to be identical to those of water at room temperature, i.e., $\varepsilon =7.1\times 10^{-10}$ F/m and $\eta =9.52\times 10^{-4}$ Pa⋅s. The correction factor, λ, was determined by fitting the computed particle trajectories to the experimentally obtained particle images.

## 4. Results and Discussion

### 4.1. Effect of Ratchet Structure

**Figure 2:**
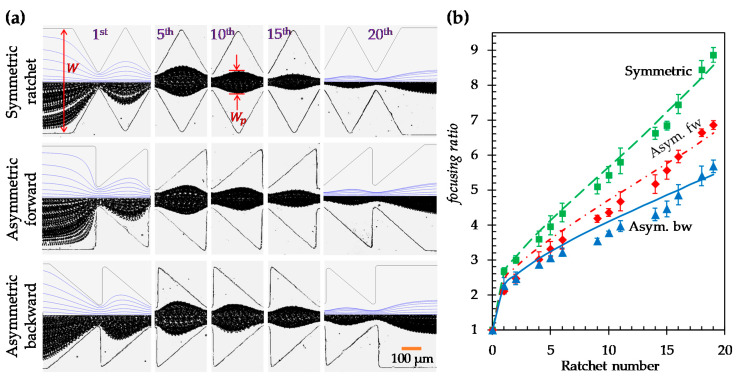
Effect of ratchet structure on the dielectrophoretic focusing of 5 µm diameter particles: (**a**) Comparison of the experimentally obtained and numerically predicted (top half of the left- and right-most images only) particle trajectories (traveling from left to right) at varying locations of the microchannels with symmetric (top row), asymmetric forward (middle row), and asymmetric backward (bottom row) ratchets, respectively; (**b**) Comparison of the experimentally measured (symbols with error bars) and numerically calculated (curves) particle focusing ratios, defined as the channel width, W, over the particle stream width, $W_{p}$ (see the highlighted dimensions in (a)), among the three ratchet structures.

Figure 2a shows the experimentally obtained top-view images of 5 µm particles in both the symmetric and asymmetric ratchet microchannels under a fixed DC electric field of 250 V/cm (specifically, a 200 V DC voltage drop averaged over the 0.8 cm long channel). For the asymmetric ratchets, the direction of the DC electric field is also switched to further study the effect of particle movement direction (with respect to the inclined surface of each ratchet) on the dielectrophoretic focusing of particles. Following our earlier study on particle trapping in an asymmetric ratchet microchannel [37], we still define the particle movement direction along which the inclined surface of each ratchet follows its normal surface as the *asymmetric forward* motion and its opposite as the *asymmetric backward* motion. To demonstrate the development of particle focusing in each of these ratchet structures, we present in Figure 2a the particle images at five different locations (specifically, at the 1st, 5th, 10th, 15th, and 20th ratchets) along the length of each ratchet microchannel. As expected, particles are gradually focused towards the channel centerline when they travel through each type of ratchet microchannel. The best particle focusing is achieved in the channel with symmetric ratchets. The worst particle focusing occurs in the *asymmetric backward* motion. These phenomena are reasonably predicted in our numerical model, where the correction factor, λ, for particle DEP in Equation (4) was set to 0.7 for all ratchet structures. This is demonstrated by the visual similarity in Figure 2a between the experimentally and numerically obtained particle trajectories at varying ratchets in every ratchet structure. Note that the numerical results are displayed for only the entrance and exit of the ratchet region in the figure. 

To quantify the ratchet structure’s effect on particle focusing, we define a dimensionless focusing ratio as the microchannel width, W, over the particle stream width, $W_{p}$ (see the highlighted dimension on the particle image in Figure 2a):

(5) $$focusing\;ratio=\frac{W}{W_{p}}$$

**Figure 3:**
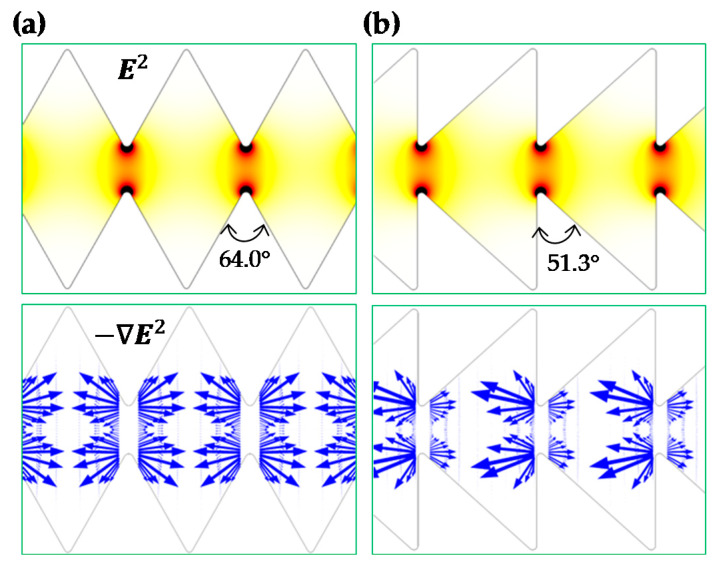
Comparison of the numerically predicted contour of electric field squared (top row), $E^{2}$ (the darker color the larger magnitude), and arrows (length proportional to the velocity magnitude) of negative dielectrophoretic particle velocity, $U_{DEP}$, in terms of $-\nabla E^{2}$ in between a symmetric (**a**) and an asymmetric (**b**) ratchet microchannel.

The comparison of the particle focusing ratios among the three ratchet structures is illustrated in Figure 2b. A good agreement between the experimental and numerical data is obtained in every ratchet structure. The focusing ratio exhibits an approximately linear (with a positive correlation) relationship with respect to the ratchet number (except for the zeroth ratchet, where particle DEP ceases). The slope of the linear trendline for the data points (excluding that at the zeroth ratchet) is approximately 0.34 for the symmetric ratchets. This value is 42% greater than the slope of the linear trendline (≈0.24) for the asymmetric forward motion and 79% greater than that (≈0.19) for the asymmetric backward motion. We attribute the strongest particle focusing effect in the symmetric ratchet microchannel to: (1) the larger opening angle, α (= 64.0°), of the ratchet tip in Equation (1) (see Figure 3a) than that (= 51.3°) in the asymmetric ratchet microchannel (see Figure 3b), and (2) the smaller discrepancy in the upstream and downstream particle dynamics as demonstrated by the symmetry of the electric field (squared) and DEP before and after the ratchet tips in Figure 3. In between the two asymmetric ratchet structures, particle DEP becomes highly asymmetric on the two sides of the ratchet in Figure 3b. Specifically, for the asymmetric forward motion, an increase in the DEP on the side of the ratchet with a normal surface to the microchannel (i.e., the upstream side of the ratchet) significantly enhances the particle deflection because it increases $\left|U_{DEP\_n}\right|$ in the numerator while decreasing the particle velocity, $U_{EK}-\left|U_{DEP\_s}\right|$, in the denominator of Equation (1). By contrast, for the asymmetric backward motion, a stronger DEP on the downstream side of the ratchet does not necessarily enhance the particle deflection because it increases both $\left|U_{DEP\_n}\right|$ in the numerator and the particle velocity, $U_{EK}+\left|U_{DEP\_s}\right|$, in the denominator of Equation (1).

### 4.2. Effect of Electric Field in the Symmetric Ratchet Microchannel

**Figure 4:**
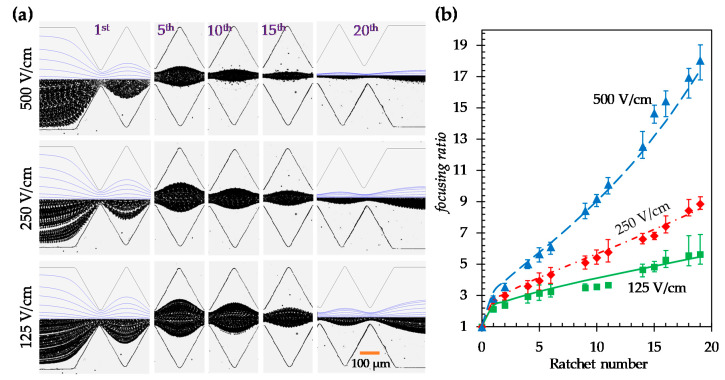
Effect of the electric field on the dielectrophoretic focusing of 5 µm diameter particles in the symmetric ratchet microchannel: (**a**) Comparison of the experimentally obtained and numerically predicted (top half of the left- and right-most images only) particle trajectories (traveling from left to right) at varying locations of the microchannel under 125 (bottom row), 250 (middle row), and 500 V/cm (top row) electric fields, respectively; (**b**) Comparison of the experimentally measured (symbols with error bars) and numerically calculated (curves) particle focusing ratios among the three electric fields.

We further study in this section and the next sections the effects of electric field and particle size, respectively, on the DC dielectrophoretic focusing of particles in the symmetric ratchet microchannel. Figure 4a shows the experimental and numerical images of 5 μm particles under 125, 250, and 500 V/cm electric fields, respectively. The correction factor, λ, for the dielectrophoretic particle velocity in the model was set to 0.7 in all three cases. As predicted by Equation (2), the particle deflection increases under a higher electric field, leading to an enhanced focusing towards the channel centerline. Figure 4b compares the experimentally measured and numerical predicted particle focusing ratios that show good agreement in every electric field. Moreover, similar to the observation in Figure 2b, the focusing ratio increases almost linearly with the number of ratchets under all three electric fields (except for the zeroth ratchet). The slopes of the linear trendlines for the particle focusing ratio, i.e., focusing ratio per ratchet, are 0.19, 0.34, and 0.78 under 125, 250, and 500 V/cm electric fields, respectively. Interestingly, the obtained values for the focusing ratio per ratchet also exhibit an approximately linear correlation with the DC electric field, which can be understood as follows. Our numerical simulation indicates that the magnitude of the streamwise dielectrophoretic velocity, $U_{DEP\_s}$, at the throat of the ratchets is no more than 10% of that of the local electrokinetic velocity, $U_{EK}$, even under the highest electric field of 500 V/cm. Further considering that the direction of $U_{DEP\_s}$ alternates before and after any pairs of ratchets, we may safely neglect its contribution to the particle deflection within one period of ratchets in Equation (2) for a symmetric ratchet microchannel, i.e.,

(6) $$deflection=\frac{2\alpha }{\left|\frac{\mu_{EK}}{\mu_{DEP}}\frac{1}{E}+\frac{2}{E}\frac{\partial E}{\partial s}\right|}\sim 2E\alpha \left|\frac{\mu_{DEP}}{\mu_{EK}}\right|$$

Thus, neglecting the action of DEP from the ratchets on the other half of the microchannel, which is equivalent to assuming that the channel width W→∞ or the particle deflection is very small compared to W, we can obtain the half-width of the particle stream as

(7) $$\frac{W_{p}}{2}\sim \frac{W}{2}-m\times deflection\sim \frac{W}{2}-2mE\alpha \left|\frac{\mu_{DEP}}{\mu_{EK}}\right|$$

where m is the number of ratchets that particles have traveled through. Then, we can rewrite the particle focusing ratio in Equation (5) as follows:

(8) $$focusing\;ratio\sim \frac{W}{W-4mE\alpha \left|\frac{\mu_{DEP}}{\mu_{EK}}\right|}$$

The focusing ratio per ratchet is hence determined as

(9) $$\begin{array}{l} focusing\;ratio\;per\;ratchet\sim \frac{W}{W-4\left(m+1\right)E\alpha \left|\frac{\mu_{DEP}}{\mu_{EK}}\right|}-\frac{W}{W-4mE\alpha \left|\frac{\mu_{DEP}}{\mu_{EK}}\right|}\\ \;\;\;\;\;\;=\frac{4WE\alpha \left|\frac{\mu_{DEP}}{\mu_{EK}}\right|}{\left(W-4\left(m+1\right)E\alpha \left|\frac{\mu_{DEP}}{\mu_{EK}}\right|\right)\left(W-4mE\alpha \left|\frac{\mu_{DEP}}{\mu_{EK}}\right|\right)}\sim \frac{4E\alpha }{W}\left|\frac{\mu_{DEP}}{\mu_{EK}}\right| \end{array}$$

Note that in this derivation, we have used the assumption of small particle deflection as compared to the channel width. Therefore, the particle focusing ratio per ratchet in Equation (8) becomes a linear function of the applied electric field. 

### 4.3. Effect of Particle Size in the Symmetric Ratchet Microchannel

**Figure 5:**
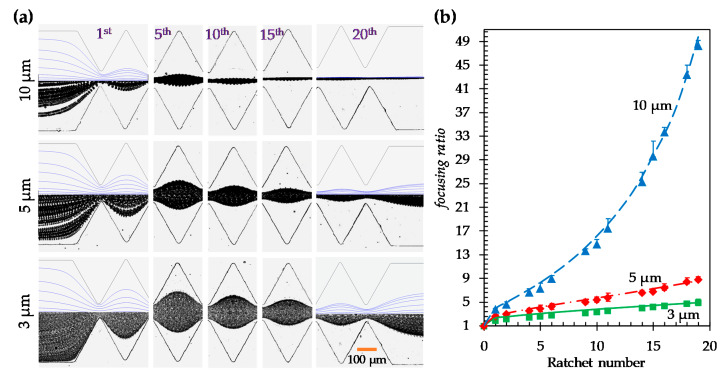
Effect of particle size on the dielectrophoretic focusing of polystyrene particles in the symmetric ratchet microchannel under a fixed DC electric field of 250 V/cm: (**a**) Comparison of the experimentally obtained and numerically predicted (top half of the left- and right-most images only) trajectories (traveling from left to right) of 3 (bottom row), 5 (middle row), and 10 µm (top row) particles, respectively, at varying locations of the microchannel; (**b**) Comparison of the experimentally measured (symbols with error bars) and numerically calculated (curves) particle focusing ratios among the three types of particles.

Figure 5a shows the experimental and numerical images of 3, 5, and 10 µm particles in the symmetric ratchet microchannel under a fixed DC electric field of 250 V/cm. The correction factor, λ, was set to 0.8, 0.7, and 0.6 for 3, 5, and 10 µm particles, respectively, in the simulation. As the dielectrophoretic mobility of particles, $\mu_{DEP}$, (see Equation (3)) is a second order function of particle size, the focusing ratio in Equation (7) should increase for larger particles because of their enhanced deflection. This is supported by the experiment and simulation in Figure 5a, where 10 µm particles attain nearly single-file focusing at the end of the ratchet region, while 3 µm particles experience only slight focusing. Figure 5b compares the experimental and numerical data of the particle focusing ratio, where good agreement is seen for all three types of particle. However, the focusing ratio for 10 µm particles exhibits an apparently nonlinear relationship with the ratchet number, though that for 3 µm particles still follows a linear trend (excluding the data at the zeroth ratchet). It may be because the $U_{DEP\_s}$ of 10 µm particles becomes comparable to $U_{EK}$, which invalidates the scaling analysis in the preceding section. In fact, the focusing ratio for 5 µm particles at 500 V/cm in Figure 4b already displays a visible deviation from the linear trendline because of the same reason. As predicted by Equation (8), the particle focusing ratio per ratchet is proportional to the magnitude of $\mu_{DEP}$ and hence a second order function of particle size. This analysis is well supported by the value of 0.16 for 3 µm particles against that of 0.34 for 5 µm particles.

### 4.4. Focusing of Yeast Cells in the Symmetric Ratchet Microchannel

**Figure 6:**
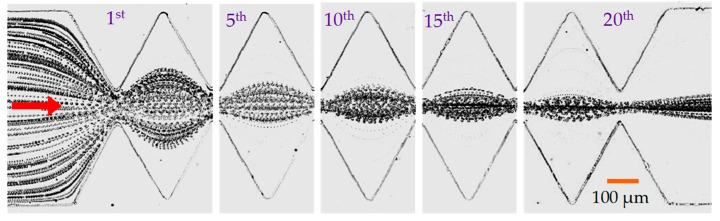
Top-view superimposed images demonstrating the development of yeast cell focusing at varying locations of the symmetric ratchet microchannel under a DC electric field of around 300 V/cm. The block arrow indicates the movement direction of cells.

To demonstrate the potential biological applications of the passive dielectrophoretic particle focusing method, yeast cells were chosen to replace 5 μm polystyrene particles in a test with the symmetric ratchet microchannel. The superimposed images in Figure 6 show the development of cell focusing along the microchannel under the application of a 250 V DC voltage (i.e., a 312.5 V/cm electric field, on average, over the entire channel length). Since the size of yeast cells is not homogenous, the observed cell focusing is slightly worse than that of 5 μm particles (see Figure 2a). The application of the DC electric field may affect the viability of yeast cells via Joule heating-induced temperature elevation [57] and/or electrical field-induced transmembrane voltage [58]. For the former, we did not notice any significant increase in the electric current through the buffer solution in the microchannel, which indicates an insignificant Joule heating effect during the focusing experiment [49]. To check the impact of the electrical shock, we conducted a viability test using trypan blue, which can stain non-viable cells blue while viable cells remain unstained. Specifically, 100 µL yeast cell suspension was taken from the outlet reservoir of the ratchet microchannel and stained with trypan blue in 1:1 ratio. A hemocytometer slide was then filled with the stained cell suspension and incubated at room temperature for 1–2 min. Live and dead cells were counted under a microscope, and the viability was calculated by dividing the number of live cells by the total number of cells. We confirmed that more than 98% of the yeast cells still remained alive after the dielectrophoretic focusing experiment.

It is worth mentioning that our group has recently demonstrated a passive focusing of particles [34] and cells [59] in a serpentine microchannel via curvature-induced DEP. Compared to that method, the current dielectrophoretic particle focusing in a ratchet microchannel has the disadvantage of drawing significantly higher electric fields around the ratchet tips, which may cause potential thermal [57] and electrical [58] issues for the sample and/or the microfluidic device as noted above. However, the current method has the capability of focusing much smaller particles because of the much stronger electric field gradients around the ratchet tips than around the corners of a serpentine microchannel. Moreover, the DEP in ratchet-like microchannels offers more diverse applications such as the focusing, concentration [35], patterning [37], electroporation [60], and separation [40] of particles or cells. It therefore has the potential to perform multiple functions in a single microfluidic device. 

## 5. Conclusions

We have performed a combined experimental, numerical, and theoretical study of the DC dielectrophoretic focusing of polystyrene particles in symmetric and asymmetric ratchet microchannels with similar dimensions. The symmetric ratchet microchannel is found to offer better particle focusing than the asymmetric one because of the larger opening angle of the symmetric ratchets. In the asymmetric ratchet microchannel, particles can attain a stronger focusing effect in the forward motion than in the backward motion because of both the asymmetry and the directional switch of particle DEP on the upstream and downstream sides of any pair of ratchets. Moreover, we have investigated the effects of electric field and particle size on the DC dielectrophoretic focusing of polystyrene particles in the symmetric ratchet microchannel. The defined dimensionless particle focusing ratio is found to increase for larger particles under higher electric fields. It also increases almost linearly with the number of ratchets, through which particles have travelled, unless the streamwise dielectrophoretic particle velocity becomes comparable to the electrokinetic velocity at the ratchet region. These phenomena can be reasonably explained by the formulae that are obtained from a theoretical analysis and may serve as a guideline for the design of ratchet microchannels in future particle focusing applications. In addition, we have demonstrated the passive dielectrophoretic focusing of yeast cells in the symmetric ratchet microchannel. The impact of DC electric field exposure on cell viability is found to be minimal under our experimental conditions. 

Compared to other *passive* focusing methods, our demonstrated DC dielectrophoretic focusing of particles and cells in ratchet microchannels has the advantages of simplicity, being free of moving parts, and being easy to integrate with other electrically-controlled microfluidic components, etc. It does not require the patterning of microelectrodes that is needed for classical AC DEP-based focusing. While it provides a much smaller throughput than fluid inertia-based hydrodynamic focusing, our electrokinetic method may find a niche application in areas that need to process small amounts of samples. Moreover, if the channel-to-constriction width ratio and/or the number of ratchets becomes sufficient large, our method has the potential to work with submicron particles or even nanoparticles that are usually very hard to control using inertial microfluidics [61]. We are currently working on how to optimize the ratchet structure for particle focusing via DC DEP.
